# PERi-operative Selective Decontamination of the digestive tract to prevent severe infectious complications after Esophageal Resection: study protocol of the PERSuaDER-trial, a multicenter randomized controlled trial in patients with primary resectable esophageal carcinoma

**DOI:** 10.1186/s13063-025-09311-w

**Published:** 2025-12-04

**Authors:** Justin G. A. Grootenhuis, Ward Seurs, Linda M. Garms, Marjan de Vries, Sander Ubels, Henri C. van Werkhoven, Camiel Rosman, Jeroen A. Schouten, Bastiaan R. Klarenbeek, Hans Van Veer

**Affiliations:** 1 Department of Surgery, Radboud University Medical Center, Nijmegen, the Netherlands; 2 Department of Thoracic Surgery, University Hospitals Leuven, Louvain, Belgium; 3 Department of Chronic Diseases and Metabolism (CHROMETA), Laboratory of Respiratory Diseases and Thoracic Surgery (BREATHE), KU Leuven, Louvain, Belgium; 4 Department of Surgery, Canisius Wilhelmina Hospital, Nijmegen, the Netherlands; 5 Julius Center for Health Sciences and Primary Care, UMC Utrecht, Utrecht University, Utrecht, The Netherlands; 6 Department of Intensive Care Medicine, Radboud University Medical Center, Nijmegen, the Netherlands; 7 Scientific Center for Quality of Healthcare (IQ Healthcare), Radboud University Medical Center, Nijmegen, the Netherlands; 8 Radboud Center for Infectious Diseases (RCI), Radboud University Medical Center, Nijmegen, the Netherlands

**Keywords:** Esophageal carcinoma, Esophagectomy, Pneumonia, Anastomotic leakage, Postoperative infectious complications, Selective decontamination of the digestive tract (SDD), Quality of life (QoL)

## Abstract

**Background:**

Esophagectomy is a highly complex surgical procedure, and it is associated with significant morbidity and mortality. The postoperative complication rate is high, with pneumonia being the most common. These are thought to arise from (micro-)aspiration of bacteria residing in the oropharyngeal and gastrointestinal (GI) tract. Selective decontamination of the digestive tract (SDD) is a prophylactic antibiotic strategy aimed at preventing these infections. SDD decolonizes the oropharyngeal and GI tract from pathogenic aerobic gram-negative rods, fungi and yeasts, while anaerobic, protective microbiota are preserved. The PERSuaDER trial aims to evaluate whether perioperative SDD can reduce postoperative pneumonia after esophagectomy.

**Methods:**

The PERSuaDER-trial is a randomized, controlled, open-label, superiority, multicenter, pragmatic, group-sequential trial aiming to include 854 patients with primary resectable esophageal carcinoma ((y)cT1-4a N0-3 M0) scheduled for transthoracic esophagectomy. The intervention group will receive SDD prophylaxis in addition to the standard care regimen. The SDD treatment comprises two oral liquids: an oral suspension containing amphotericin B and an oral solution containing both colistin sulfate and tobramycin. Participants are required to swallow the SDD suspension four times a day for 1 week, starting 3 days prior to the surgical procedure, with two doses on the day of surgery. All other aspects of care are identical to those provided to the control group (standard care without SDD). Participants will be asked to keep a diary and fill out quality of life, medical consumption, and productivity cost questionnaires. Interim analyses will take place after 30% and 60% of the participants have completed follow-up of 30 days after surgery, and after completion or termination of the trial.

**Discussion:**

It is hypothesized that the addition of SDD to the standard peri-operative care in esophageal cancer surgery will result in a reduction in the incidence of postoperative pneumonia. Furthermore, the trial will evaluate the impact of SDD on postoperative (infectious) complications and quality of life, and its cost-effectiveness.

**Trial registration:**

The PERSuaDER trial is registered in ClinicalTrials.gov (NCT05865743) and the European Clinical Trials Information System (CTIS) (EU Trial number: 2023-504144-33), Trial authorization date: 25.03.2024, protocol version 1.1, date: 03.03.2024.

## Administrative information

Note: the numbers in curly brackets in this protocol refer to SPIRIT checklist item numbers. The order of the items has been modified to group similar items (see http://www.equator-network.org/reporting-guidelines/spirit-2013-statement-defining-standard-protocol-items-for-clinical-trials/).

**Table:** 

Title {1}	PERi-operative Selective Decontamination of the digestive tract to prevent severe infectious complications after Esophageal Resection: study protocol of the PERSuaDER-trial, a multicenter randomized controlled trial in patients with primary resectable esophageal carcinoma.
Trial registration {2a and 2b}.	ClinicalTrials.gov registration, NCT05865743 and the European Clinical Trials Information System (CTIS) (Eu Trial number: 2023–504144-33), Trial authorization date: 25.03.2024.
Protocol version {3}	Protocol version 1.1, date 03.03.2024
Funding {4}	The PERSuaDER trial is funded by a BeNeFIT grant, which is a collaboration between ZonMw (Care Research Netherlands/Medical sciences) and KCE (Belgian Health Care Knowledge Centre). The funding sources have no role in conduction, analysis, interpretation or publication of this study.
Author details {5a}	§ Contributed equally 1 Department of Surgery, Radboud University Medical Center, Nijmegen, the Netherlands. 2 Department of Thoracic Surgery, University Hospitals Leuven, Leuven, Belgium. 3 Department of Chronic Diseases and Metabolism (CHROMETA), Laboratory of Respiratory Diseases and Thoracic Surgery (BREATHE), KU Leuven, Leuven, Belgium. 4 Department of Surgery, Canisius Wilhelmina Hospital, Nijmegen, the Netherlands. 5 Julius Center for Health Sciences and Primary Care, UMC Utrecht, Utrecht University, Utrecht, The Netherlands. 6 Department of Intensive Care Medicine, Radboud University Medical Center, Nijmegen, the Netherlands. 7 Scientific Center for Quality of Healthcare (IQ healthcare), Radboud University Medical Center, Nijmegen, the Netherlands. 8 Radboud Center for Infectious Diseases (RCI), Radboud University Medical Center, Nijmegen, the Netherlands.
Name and contact information for the trial sponsor {5b}	Radboud University Medical Center Geert Grooteplein Zuid 10 6525 GA Nijmegen The Netherlands
Role of sponsor {5c}	The sponsor is responsible for setting up the trial, trial coordination, data collection and analysis and submitting the final manuscript for publication.

## Introduction

### Background and rationale {6a}

The incidence of esophageal carcinoma has increased considerably within the last thirty years worldwide and is expected to keep on rising [1, 2]. This change will likely lead to an increase in the number of esophagectomies that will be performed. Despite the implementation of minimally invasive surgical techniques, these surgeries are regularly accompanied by infectious complications, with postoperative pneumonia being among the most common (up to 40%) and anastomotic leakage (10–20%) the most dreaded [3–6]. These complications lead to an increase in hospitalization time, healthcare costs, and decreased long-term survival [7–9].

Postoperative pneumoniae are thought to arise from (micro-)aspiration of bacteria residing in the oropharyngeal and gastrointestinal (GI) tract [10]. Besides causing respiratory infections, these pathogens might also be able to induce local inflammation and abscess formation at the level of the esophago-enteric anastomosis, resulting in anastomotic dehiscence and eventually anastomotic leakage [11]. Therefore, it is hypothesized that eliminating these pathogens prior to surgery and during recovery potentially decreases the risk of postoperative infections and subsequent anastomotic leakage.

Aiming to prevent postoperative complications, some hospitals in the Netherlands already use a perioperative prophylactic antibiotic strategy that reduces GI colonization with aerobic gram-negative rods and yeasts, while preserving anaerobic microbiota [12]. This strategy, named selective decontamination of the digestive tract (SDD), consists of three oral, non-absorbable antimicrobial agents (colistin, tobramycin, and amphotericin B) that are administered four times daily as an enteral suspension [13]. SDD is associated with a reduced risk of ventilator-associated pneumonia in intensive care unit (ICU) patients [14].

A recent retrospective cohort study including 496 patients undergoing esophagectomy, showed that perioperative application of SDD was associated with a reduction in postoperative pneumonia (36.9% vs 20.1%, adjusted odds ratio (OR) 0.40 (0.23–0.67)) and anastomotic leakage (19.9% vs. 10.6%, adjusted OR 0.46 (0.26–0.84)) following esophageal surgery [15]. However, to date, prospective evidence supporting the use of SDD in the prevention of postoperative pneumonia after esophageal surgery is limited [16–20]. By evaluating the effect of SDD on infectious complications after esophagectomy, the PERSuaDER-trial aims to establish the evidence to implement SDD into the perioperative care of esophageal cancer surgery.

### Objectives {7}

The primary objective of the PERSuaDER trial is to evaluate the effect of SDD on infectious complications after esophagectomy, focusing on the prevention of pneumonia. Based on the afore mentioned findings, the hypothesis is that SDD will decrease the rate of post-operative pneumonia by 40% [15]. Secondary objectives are to determine additional benefits such as reduction of other infectious complications including anastomotic leakage, quality of life during the recovery period and evaluation of the risks of SDD. To support broader implementation, patient diaries will be used to collect data on patient preference and adherence, which are critical factors for the broader implementation of SDD. Additionally, a cost-effectiveness analysis will be conducted.

### Trial design {8}

**Fig. 1:**
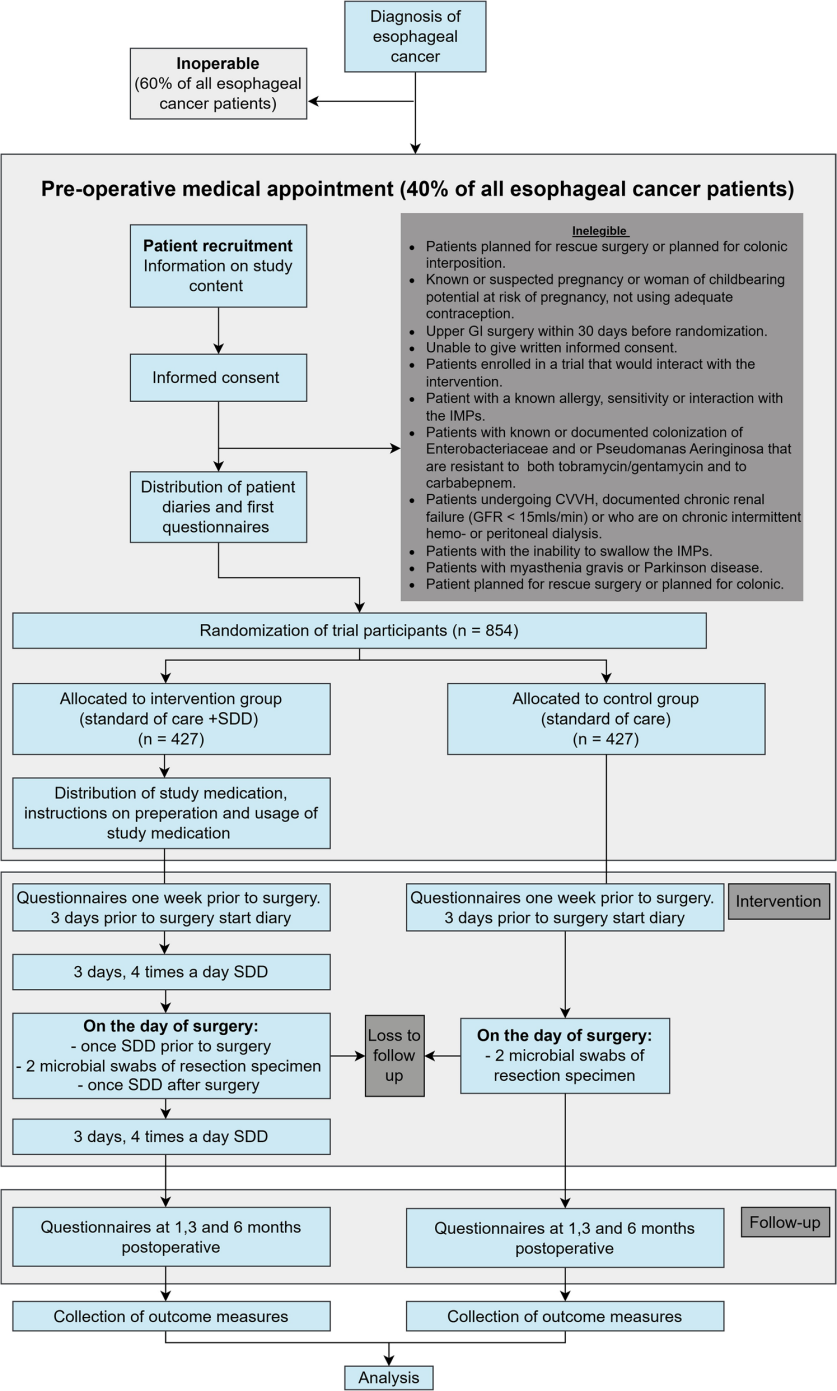
Flowchart PERSuaDER-trial

The PERSuaDER trial has been designed as an open-label, superiority, multicenter, binational, randomized controlled trial. Patients will be randomly allocated (1:1) to a control cohort receiving regular care or an intervention cohort, receiving standard care plus SDD. The trial can be classified as pragmatic, as it aims to estimate the effect of SDD under normal clinical conditions. Consequently, eligibility criteria are well aligned with intended use in daily practice, and a placebo will not be used (Fig. 1).

## Methods: participants, interventions, and outcomes

### Study setting {9}

The study will be conducted in Dutch and Belgian hospitals, specialized in the surgical treatment of esophageal cancer. These centers comprise both academic medical centers and non-academic hospitals. A list of study sites can be obtained from https://persuadertrial.org/.

### Eligibility criteria {10}

All consecutive adult patients with primary resectable esophageal carcinoma planned to undergo transthoracic esophagectomy at one of the participating Dutch and Belgian centers will be asked to participate.

#### Inclusion criteria

To be eligible to participate in this study, a subject must meet all the following criteria. First, a patient must have a diagnosis of primary esophageal adenocarcinoma or squamous cell carcinoma (cT1b-4a, N0-3, M0). Second, the tumor must be located in the mid or distal esophagus or at the level of the gastroesophageal junction. Third, the patient must be scheduled to undergo transthoracic esophagectomy with curative intent and planned for esophageal reconstruction with a gastric conduit or jejunal interposition. Participants can be included with the intention of primary surgical care or within a peri-operative chemotherapy or neo-adjuvant chemoradiotherapy schedule. Lastly, the patient must be at least 18 years old and capable of providing written informed consent.

#### Exclusion criteria

Patients will be excluded if they are planned for salvage surgery or reconstruction with colonic interposition, or have undergone resectional upper GI surgery within 30 days before randomization. Furthermore, we exclude patients who are unable to understand the study information and study instructions and give informed consent, patients who are enrolled in a trial that would interact with the intervention, patients with a known allergy, sensitivity, or interaction to the investigational medicinal product, patients who have chronic renal failure (GFR < 15mls/s) or undergo renal replacement therapy, patients with pre-existing degenerative neuromuscular diseases, women of childbearing potential not using adequate contraception, patients who are known to be colonized with or have infections caused by microorganisms resistant to tobramycin or colistin or against carbapenems, and patients with the inability to swallow the SDD.

Patients will not be excluded on the basis of old age criteria, ASA (American Society of Anesthesiology) classification or Charlson Comorbidity Index. If patients are deemed fit for surgery, meet the inclusion criteria, and none of the exclusion criteria, they are eligible to participate in the study.

### Who will take informed consent? {26a}

The treating physician will approach all patients undergoing an esophagectomy for esophageal cancer about the possibility of participating in the PERSuaDER-trial. If the potential participant has consented to be contacted by members of the research team, they will be informed about the study, and additional information regarding the trial will be provided. After the patient has read the patient information brochure, any remaining questions will be answered. Once the patient decides having had sufficient time to consider his or her decision and all questions have been answered satisfactorily, the participant will subsequently be asked to return their signed consent in the provided return envelope. Once the signed informed consent forms are received, patients will be screened for the inclusion and exclusion criteria, randomized, followed by the distribution of questionnaires, a diary, and, if allocated to the intervention group, study medication.

### Additional consent provisions for collection and use of participant data and biological specimens {26b}

The informed consent obtained from the participants will cover peri-operative data collection including the collection and analysis of the microbial swabs as part of an efficacy assessment of the intervention. Through additional questions participants are questioned for consent to share patient-reported outcome measures with the Prospective Observational Cohort Study of Oesophageal-gastric cancer Patients (POCOP) registry and to contact the participant for future studies.

### Interventions

#### Explanation for the choice of comparators {6b}

Patients will be informed that participation in the PERSuaDER trial means that they will either receive standard care without SDD or standard care with SDD.

### Intervention description {11a}

#### SDD

Patients randomized towards the intervention arm will receive SDD medication with instructions from the hospital pharmacy or research staff. The SDD treatment comprises two oral liquids: an oral suspension (5 mL) containing amphotericin B (100 mg/mL), followed by an oral solution (another 5 mL) containing both colistin sulfate (20 mg/mL) and tobramycin (16 mg/mL). Patients are required to take the SDD four times a day for 1 week, starting 3 days prior to the surgery. On the day of surgery, intake is limited to an early morning and a late evening dose. Patients will be asked to hand in the empty vials of the SDD upon hospital admission to monitor therapy compliance and drug accountability. Patients in the control group will not receive a placebo.

### Criteria for discontinuing or modifying allocated interventions {11b}

Subjects can leave the study at any time for any reason if they wish to do so without any consequences. The investigator can decide to withdraw a subject from the study intervention for urgent medical reasons.

### Strategies to improve adherence to interventions {11c}

Patient will be reminded by the local research staff to start with study medication 3 days prior to the operation date. A daily check by research staff will take place for compliance but only during admission; in the three preoperative days no check will be performed apart from the reminder prior to the start, as not to interfere with the pragmatic nature of the trial.

### Relevant concomitant care permitted or prohibited during the trial {11d}

All medication considered necessary for the patient’s welfare will be continued. Relevant concomitant medication, such as anticoagulants and corticosteroids, will be recorded within the eCRF. The use of sucralfate is prohibited as it is able to bind to components of the SDD and therefore reduces its effectiveness. If necessary, sucralfate can be substituted with an agent that has a different mechanism of action.

### Provisions for post-trial care {30}

The sponsor/investigator has a liability insurance that is in accordance with article 7 of the WMO (Dutch: “Wet Medisch-Wetenschappelijk Onderzoek met Mensen”; English: Act/law on Medical Research Involving Human Subjects). The sponsor has an insurance that is in accordance with the legal requirements in The Netherlands (article 7 WMO). This insurance provides cover for damage to research subjects through injury or death caused by the study. The insurance applies to the damage that becomes apparent during the study or within 4 years after the end of the study. For the Belgian centers, the insurance provided by the Belgian Coordinating Center will be covering no-fault liability for Belgian participants.

### Outcomes {12}

#### Primary outcome

The primary outcome parameter is the cumulative incidence of postoperative pneumonia within 30 days after surgery. Pneumonia will be defined by the following criteria: positive sputum culture or the presence of a new progressive radiographic infiltrate plus at least two of the following clinical features: fever > 38.5 °C, leucocytosis (> 11.0*10^9/L) or leukopenia (< 4.0*10^9/L), or purulent secretions.

#### Secondary outcomes

Secondary endpoints include the incidence of postoperative complications, such as anastomotic leakage based on Esophageal Complications Consensus Group (ECCG) criteria, and the rate of reoperation within 30 days following surgery [21]. Complications will be graded according to the modified Clavien-Dindo classification [22]. Other secondary endpoints are postoperative length of stay in the hospital and the intensive care unit (ICU), as well as readmissions within 6 months after surgery, mortality, quality of life (QoL), direct and indirect costs, medication adherence, and side effects.

#### Microbial assessment

Two microbial samples will be taken from the esophageal resection specimen (one from the proximal and one from the distal part) for culture in the control as well as in the intervention group to evaluate the effectiveness and compliance of SDD application.

#### Questionnaires and diaries

Patients in both the intervention and control arms will be asked to keep a diary during the perioperative period. This allows us to explore potential side effects, therapy adherence and real-life experiences of patients. Quality of Life (QoL) will be measured by three validated questionnaires (EORTC OG25, QLQC30 and EuroQol-5D-5L) provided to patients 1 week before surgery, and at 30 days, 3 months, and 6 months postoperatively [23, 24]. We will estimate direct and indirect health-related costs with the help of a medical consumption and productivity cost questionnaire developed by the iMTA [25, 26]. These questionnaires will be provided to the participants together with the QoL questionnaires at 3 and 6 months postoperatively.

#### Participant timeline {13}

Participant timeline is outlined in Table 1.

**Table 1:** Schedule of assessments and study procedures

	Study period										
	**1–6 weeks until surgery**	**D-3**	**D-2**	**D-1**	**D0**	**D1**	**D2**	**D3**	**D30**	**D90**	**D180**
	Outpatient clinic	Home		Hospital					Home		
Screening study eligibility	x										
Advising pregnancy test if applicable	x										
Information on PERSuaDER, study content and consent	x										
Collection informed consent	x										
Randomization	x										
Dispersion and instruction on intervention medication	x										
SDD intervention		x	x	x	x	x	x	x			
Patient diary		x	x	x	x	x	x	x			
Resectional specimen swabs					x						
Pneumonia assessment									x	x	x
Other complications assessment*									x	x	x
Medication adherence assessment									x		
QoL questionnaires	x								x	x	x
Other secondary outcome assessment*									x	x	x
(S)AE, SUSAR assessment **		x	x	x	x	x	x	x	x	x	x

* Other secondary outcomes: postoperative length of stay, the intensive care unit (ICU) length of stay, re-admissions, cost assessment. ** As described in the “Adverse event reporting and harms {22}” section*. QoL*, Quality of Life; *(S)AE*, (Serious) Adverse Event; *SUSAR*, Suspected Unexpected Serious Adverse Event

### Sample size {14}

Based on contemporary registries and literature, we assume an incidence of postoperative pneumonia in the control group of approximately 22% [27]. The sample size calculation is based on the primary objective of the trial to demonstrate that the intervention will result in a clinically relevant decrease of postoperative pneumonia by 40%. This would convey to an incidence of 13% (superiority) with 90% power and a two-sided alpha of 0.05. A group-sequential design is used with two interim analyses at 30% and 60% and two-sided asymmetric bounds (Hwang-Shih-DeCani spending function) for superiority and futility. The calculated sample size convenes to a total sample size of 814 study participants (407 per arm). Despite the short study intervention, some early deaths may occur and surgeries may be canceled in this time frame after randomization. Therefore, we anticipate a 5% loss to follow-up rate and will add 20 additional subjects to each group to compensate for this. As such, a total sample size of 854 participants will be required.

### Recruitment {15}

To recruit 854 patients for the study at a realistic, expected recruitment rate of ~35%, we will recruit patients for 39 months to draw from a source population of approximately 2681 patients. Therefore, we are likely to recruit the planned number of patients from the defined source population. In case of stagnating inclusion, other centers with the intention to participate will be added to this study by amendments.

### Assignment of interventions: allocation

#### Sequence generation {16a}

After confirmation of eligibility and completion of IC, randomization will be performed by the local research staff by using the built-in randomization module; the sequence (concealed within castoredc.com) will be initiated by the site study data manager, independent of the investigators and clinical staff using block randomization with variable block size, stratified by the three main confounding factors in esophageal surgery: hospital, site of anastomosis, and Charlson Comorbidity Index. Participants are allocated in a 1:1 ratio to receive either usual care alone or usual care plus the SDD treatment policy.

### Concealment mechanism {16b}

Randomization will be performed by the local research staff through the built-in randomization module in Castor EDC (www.castoredc.com), and the sequence (concealed within Castor EDC) will be initiated by the study data manager, independent of the investigators and clinical staff.

### Implementation {16c}

Eligible patients will be identified at the outpatient clinic of the surgeon. Patients will be informed about the goals of the study, its risks and benefits after which informed consent will be obtained. The intervention will be assigned by the local research staff through a randomization program (Castor EDC).

## Assignment of interventions: blinding

### Who will be blinded {17a}

As an open label study, allocation of the intervention will not be blinded. Analysis of the outcome parameters will be blinded.

### Procedure for unblinding if needed {17b}

N/A as an open label study, the allocation of the intervention will not be blinded. The analysis of the outcome parameters will be blinded.

### Data collection and management

#### Plans for assessment and collection of outcomes {18a}

During the outpatient visit at which inclusion takes place, the patient will be assigned an anonymized participation number. The key needed to identify the patient data is held by the Principal Investigator (PI) of the participating sites. During the trial, data will be collected by delegated research personnel, who are listed in the delegation log. Data for the primary, and most secondary endpoints will be collected from the patient’s medical records. Data on secondary endpoints regarding quality of life and other patient-derived outcome measures (i.e., patient diary) will be collected through questionnaires (paper-based or through CastorEDC). Data on IMP accountability will be gathered from patient medical records, patient diary, and a dedicated IMP-accountability form. This data will be entered into the electronic case report forms (eCRF). Data such as source documents will be maintained at the participating site for 25 years after the end of this study, conforming to European Union Clinical Trials Regulation (EU-CTR).

### Plans to promote participant retention and complete follow-up {18b}

To ensure participant retention and complete follow-up, a commitment has been agreed upon for the local researchers. They will contact the patients regarding the completion of the questionnaires throughout the follow-up period (Fig. 1). Patients who have enrolled in the study are at liberty to withdraw from study participation at any moment. Participants that have withdrawn from the study will not be replaced. Participants who wish to drop out of the study during follow-up will be analyzed according to the intention-to-treat principles.

### Data management {19}

The study will utilize eCRF for data collection. All data will be collected and processed in accordance with the General Data Protection Regulation (EU) 2016/679. All site staff will be trained on correct eCRF completion. Study subjects will be identified only by their unique subject number which will be used in all correspondence and further administration in the study database. The investigator and the investigation site team shall maintain patient confidentiality in all documentations. An identification log will be kept of all subject numbers linked to corresponding participants. The list will remain at the participating site. Data validation will be performed by checking the eCRFs with the medical records of all trial patients on the primary outcome by the monitor. Discrepancies will be discussed with the study nurse or site investigator. Study-related documents, patient records, signed informed consent forms, and source documents will be maintained at the participating site for 25 years after the end of this study, conform EU CTR. Source data will be entered in the online database Castor and afterwards exported for the analyses.

### Confidentiality {27}

Information obtained during the study will be regarded as confidential. The investigator and all members of the study team agree not to disclose or publish such information in any way to any third party without prior written permission from the principal investigator, which will not be unreasonably withheld, except as required by law. The investigator will take all measures to ensure patient confidentiality.

### Plans for collection, laboratory evaluation and storage of biological specimens for genetic or molecular analysis in this trial/future use {33}

Two microbial samples will be taken (using CE (conformité européenne) marked IVD’s (invitro diagnostics)) from the esophageal resection specimen (one proximal and one from the distal part) for culture to evaluate effectiveness and compliance of SDD application in control as well as in the intervention group. All responsible laboratories have ISO (International Organization for Standardization) 15189 certification. After analyses the samples are destroyed according to local laboratory protocols.

### Statistical methods

#### Statistical methods for primary and secondary outcomes {20a}

The primary analysis will be according to intention to treat, including all randomized participants. Per protocol analyses will be performed in patients that received SDD or no SDD in accordance with the randomized allocation. For the analysis of the primary outcome, we will use Cox regression (cause-specific hazard ratio which defines the effect on the daily hazard), with censoring for death and putative loss to follow-up, and we will adjust for the stratification variables by including these as strata in the regression. A sensitivity analysis will be performed using Fine and Gray regression (sub distribution hazard ratio which defines the effect on cumulative incidence) considering mortality as competing events and censoring for putative loss to follow-up, also including the stratification variables as strata.

Secondary outcomes will be analyzed using logistic regression (all-cause mortality and rate of re-operation), time-to-event analysis (incidence using Cox regression and cumulative incidence using Fine and Gray regression), or linear regression (length of stay and quality of life), as appropriate.

### Interim analyses {21b}

We will use a group-sequential design for the analysis of the primary endpoint, based on asymmetric testing design (superiority and futility). Analysis of the primary endpoint will take place after 30% (*N* = 257) and 60% (*N* = 513) of the patients have been included. If these interim analyses lead to an unambiguous conclusion on the effectiveness or futility of the intervention, this may cause an adjustment or early termination of the trial.

### Methods for additional analyses (e.g., subgroup analyses) {20b}

Explorative subgroup analyses will be performed to assess differences in efficacy between men and women and age groups.

### Methods in analysis to handle protocol non-adherence and any statistical methods to handle missing data {20c}

The amount of missing data will be described. Missing data in variables that are planned to be included in multivariable models will be imputed using multiple imputation. Missing outcome variables and missing data in planned subgroup variables will not be imputed.

### Plans to give access to the full protocol, participant-level data and statistical code {31c}

The study protocol is made available to the public on ClinicalTrials.gov under reference https://clinicaltrials.gov/study/NCT05865743.

### Oversight and monitoring

#### Composition of the coordinating center and trial steering committee {5d}

At both coordinating centers, a PhD candidate and a research coordinator oversee the trial’s daily operations under the supervision of two principal investigators. Biweekly evaluations are conducted with the trial steering committee, with additional consultations available throughout the week. Additionally, an adjudication committee has been established to analyze the primary endpoint. This adjudication committee is composed of 3 unbiased non-blinded experts that will meet after 30% and 60% of participants have completed follow-up of 30 days after surgery, and after completion or termination of the trial.

### Composition of the data monitoring committee, its role and reporting structure {21a}

To monitor safety, futility and efficacy, an independent Data Safety Monitoring Board (DSMB) is appointed. A DSMB charter is created according to DAMOCLES guidelines [28]. The DSMB will have access to the gathered primary and secondary endpoint data. The DSMB will review the cause of death from all deceased patients to evaluate possible relations to the study intervention. The board consists of a statistician, an intensivist/toxicologist and a surgeon to ensure a comprehensive assessment of the data from multiple clinical and methodological perspectives. After every meeting, the DSMB will report to the trial steering committee to discuss results. The DSMB’s advice will be sent to the Principal Investigators of the sponsor institute supervising the trial in the Netherlands and the Belgian coordinating center. Should these PIs decide not to fully implement the advice of the DSMB, the sponsor will send the advice to the reviewing METC, including a note to substantiate why (part of) the advice of the DSMB will not be followed.

### Adverse event reporting and harms {22}

#### Adverse events (AEs)

Adverse events are defined as any untoward medical occurrence in a subject to whom a medicinal product is administered that does not necessarily have a causal relationship with this treatment. These AEs will be captured with CastorEDC, our electronic case report form (eCRF), as well as patient-reported outcome measures (PROMs) such as patient diaries and questionnaires.

#### Serious adverse events (SAEs)

A serious adverse event is any untoward medical occurrence or effect that results in death, is life threatening (at the time of the event), requires hospitalization or prolongation of hospitalization, results in persistent or significant disability or incapacity, or is a congenital anomaly or birth defect. Furthermore, an important medical event may be considered a Serious Adverse Event (SAE) when it potentially jeopardizes the subject. This determination is based on appropriate medical judgment and may require medical or surgical intervention to prevent serious outcomes.

To account for complications that are specific to the cancer treatment offered to our patients, and differentiate these, we defined context-specific serious adverse events as SAEs that are known complications of the disease, treatment or study intervention.

#### Suspected unexpected serious adverse reactions (SUSARs)

Adverse reactions are SUSARs if the following three conditions are met. First, the event must be serious. Second, there must be a certain degree of probability that the event is a harmful and undesirable reaction to the medicinal product under investigation, regardless of the administered dose. Last, the adverse reaction must be unexpected; that is to say, the nature and severity of the adverse reaction are not in agreement with the product information.

#### Reporting of AEs

Esophageal surgery is associated with considerable morbidity and mortality; therefore, numerous AEs and SAEs will occur within our study. It is clear that reporting all AEs has no added value and will not enhance patients’ safety. Therefore, we agreed to only note in the eCRF the AEs listed in the ECCG consensus on complications of esophageal resection from Clavien-Dindo score 3 onwards. AEs not documented in the above-mentioned ECCG list will be entered in the eCRF regardless of Clavien-Dindo score.

#### Reporting of SAEs/SUSARs by the investigator to the sponsor

The context-specific serious adverse events need to be reported in the AE section in the eCRF within 72 h after becoming aware (to avoid an incomplete annual safety report). If there occur SAEs/SUSARs that cannot be reasonably expected from the surgical intervention, study intervention or the disease for which the surgical intervention is planned, the investigator will report to the sponsor within 24 h.

#### Reporting of SUSARs to the EUDRAVigilance database

The sponsor takes responsibility for the electronic reporting of SUSARs in EUDRAVigilance without delay. The period for the reporting of SUSARs to the EMA by EUDRAVigilance will take into account the seriousness of the reaction and will be as follows: First, in the case of fatal or life-threatening SUSARs, as soon as possible and in any event not later than 7 days after the sponsor became aware of the reaction. Second, in the case of non-fatal or non-life-threatening SUSARs, not later than 15 days after the sponsor became aware of the reaction. Third, in the case of a SUSAR that was initially considered to be non-fatal or non-life threatening but which turns out to be fatal or life-threatening, as soon as possible and in any event not later than 7 days after the sponsor became aware of the reaction being fatal or life-threatening. Where necessary to ensure timely reporting, the sponsor may, in accordance with section 2.4 of Annex III, submit an initial incomplete report followed up by a complete report (CTR: Article 42 [2]).

### Frequency and plans for auditing trial conduct {23}

At any given point during the study, the trial can be selected for audit. There is no predefined schedule for audits and inspections.

### Plans for communicating important protocol amendments to relevant parties (e.g., trial participants, ethical committees) {25}

If any substantial modifications to the original documents are made we will submit these via CTIS. After approval the DSMB, Monitor, Trial Steering Committee, and the local principal investigators will be notified. If necessary updated documents will be provided for the participating hospitals and adjusted on the trial website.

### Dissemination plans {31a}

The results of the PERSuaDER trial will be fully disclosed through publication in a peer-reviewed journal and through presentations at national and international scientific conferences.

## Discussion

Esophagectomies are often followed by (infectious) complications, especially pneumonia. To reduce the risk of pneumonia, some hospitals in the Netherlands already use SDD. However, the scientific evidence remains limited. The large studies demonstrating the safety and efficacy of SDD in reducing infectious complications and mortality were never conducted in esophageal patients. These trials primarily involved patients in intensive care units or those undergoing elective (mostly lower) gastrointestinal surgery [14, 29, 30]. To date, little evidence exists regarding the perioperative use of SDD in esophageal cancer surgery. The few studies addressing this population are generally observational, vary in SDD composition and duration and are often small or of lower quality.

We designed a large, open-label randomized controlled trial comparing standard care with SDD to standard care without SDD, to find the much-needed evidence on the efficacy of SDD in patients undergoing esophageal cancer surgery. We selected the most widely used SDD formulation, combining amphotericin-B, colistin and tobramycin to target not only the most common bacterial pathogens, namely gram-negative rods, but also yeasts. Our study does have some limitations. A key point to consider is that no placebo will be used in our trial. Patients receiving SDD in addition to standard care will be compared to a control group receiving standard care without a placebo. Incorporating a placebo in this large, investigator-initiated drug trial was deemed impractical, as a significant portion of the intervention occurs at home without study staff supervision. This design reflects a real-life scenario, in contrast to the potential selection bias that may arise in placebo-controlled trials. However, the absence of a placebo increases the risk of biased outcome assessment. To mitigate this, we have implemented objective criteria for the primary endpoint. Furthermore, global shortages of SDD components may require adjustments to the composition of our investigational medicinal product (IMP). However, a suitable alternative is available, and such modifications also occur in clinical practice. Therefore, the intervention remains well-defined and reflective of real-world conditions, making the intervention sufficiently well-defined.

The PERSuaDER-trial aims to provide a robust and prospective evaluation of perioperative SDD as a protective strategy to reduce postoperative pneumonia in esophageal cancer surgery. The results of this trial could play a crucial role in guiding the decision-making process regarding the implementation of this off-patent, widely available prophylactic strategy, potentially resulting in significant health benefits and a substantial reduction in healthcare costs.

### Trial status

Protocol version 1.1, March 3rd, 2024. The trial has been recruiting since October 21 st, 2024, with recruitment expected to conclude in December 2027.

## Data Availability

The study protocol, derived data, and statistical analysis code will be made available upon request.

## Abbreviations

AE: Adverse event
ASA: American Society of Anesthesiology
CCI: Charlson Comorbidity Index
CE: Conformité Européenne
CTIS: Clinical Trials Information System
DSMB: Data Safety Monitoring Board
ECCG: Esophageal Complications Consensus Group
eCRF: Electronic Case Report Form
EMA: European Medicine Agency
EU CTR: European Union Clinical Trials Regulation
EU: European Union
GFR: Glomerular filtration rate
GI: Gastrointestinal
ICU: Intensive care unit
IMP: Investigational Medicinal Product
ISO: International Organization for Standardization
IVD: Invitro diagnostic
KCE: Belgian Health Care Knowledge Centre
MERC: Medical Ethics Review Committee
METC: Medical Ethics Committee; in Dutch: Medisch Ethische Toetsings Commissie
OR: Odds ratio
POCOP: Prospective Observational Cohort Study of Oesophageal-gastric cancer Patients
POD: Postoperative day
PROM: Patient-reported outcomes measurements
QoL: Quality of life
RCT: Randomized controlled trial
SAE: Serious adverse event
SDD: Selective decontamination of the digestive tract
SUSAR: Suspected unexpected serious adverse reaction
UMC: University Medical Center
WMO: Medical Research Involving Human Subjects Act; in Dutch: Wet Medisch-Wetenschappelijk Onderzoek met Mensen
ZonMw: Care Research Netherlands/Medical sciences
